# From crisis to capacity: Lessons learned from youth e-mentoring during the COVID-19 pandemic

**DOI:** 10.1016/j.chbr.2024.100400

**Published:** 2024-05

**Authors:** Kate Wright, Deborah K. Levine, Maritza Salcido, Michael Garringer, Tselza Almendra, Alicia Bazell, Michelle R. Kaufman

**Affiliations:** a Johns Hopkins Bloomberg School of Public Health, 624 N. Broadway, Baltimore, MD, 21205, USA; b Johns Hopkins Bloomberg School of Public Health, 447 43rd Street, Oakland, CA 94609, USA; c University of Erlangen-Nuremberg, Schloßplatz 4, 91054, Erlangen, Germany; d MENTOR, 201 South Street, Suite 615, Boston, MA, 02111, USA; e Johns Hopkins Bloomberg School of Public Health, 3743 S. Ferntower Ave, West Covina, CA, 91792, USA

**Keywords:** Youth mentoring, e-mentoring, COVID-19, Adult mentors, Youth mentees, Virtual mentoring

## Abstract

The COVID-19 pandemic and associated need for social isolation left in-person youth mentoring programs scrambling to keep mentees and mentors connected, and many programs turned to e-mentoring. To better understand the transition period and to inform e-mentoring practice in a post-COVID world, this study explored the experience of mentoring programs shifting to e-mentoring during the first year of the pandemic. Seven remote focus group discussions were conducted with twenty-three staff members from twenty U.S. youth mentoring organizations that used the iCouldBe e-mentoring platform during Spring/summer 2020 or Fall/Winter 2020–2021. Thematic content analysis was used to uncover insights from the data. E-mentoring was successful overall for keeping mentees and mentors in touch, especially for matches with a strong connection before the pandemic. Zoom and text messaging were the most used virtual communication methods. Programs faced many challenges but also experienced unexpected positives, including a strong interest in future e-mentoring implementation. Participants recommended that programs interested in e-mentoring start small and with intention; they also requested a central website with e-mentoring support and ways to connect with other programs and mentors. Although the literature on e-mentoring remains limited, this study contributes a picture of e-mentoring success even during a global crisis.

## Highlights

• In-person youth mentoring programs faced many challenges when they transitioned to e-mentoring during COVID-19.
• Unexpected positives included a strong interest in future implementation of e-mentoring.
• Mentoring program staff recommended that programs interested in e-mentoring start small and with intention.
• Staff requested a central mentor website with e-mentoring support and ways to connect with others.

## 1 Introduction

Youth mentoring involves a young person who receives consistent guidance and support from a trusted adult mentor through one-to-one or small group engagement, outside of a professional counseling or therapy role. The mentee/mentor relationship aims to encourage positive development and long-term success for the mentee (Dubois & Karcher, 2013; Raposa et al., 2019, Raposa et al., 2019). Most mentoring programs operate primarily in person (Garringer et al., 2017). When the COVID-19 pandemic emerged in the United States in March 2020, much of the country issued a stay-at-home order, during which time people spent most of their days inside to reduce the risk of disease transmission. This quarantine brought school closures and resulted in social isolation. As a result, this created enormous distress for the youth mentoring community as programs struggled to keep relationships between mentors and mentees intact during a time when they could not see each other face-to-face (MENTOR, 2020). Many mentoring programs began contemplating the idea of shifting to e-mentoring as an immediate response to the needs of their youth.

### 1.1 E-mentoring and iCouldBe

E-mentoring includes intentional and consistent interaction between the mentee and the mentor through the use of technology. Virtual communication may be the only means of interaction, or it may supplement in-person interactions (Garringer et al., 2019; Kaufman, 2017). Before the pandemic, e-mentoring was typically only used for specific youth populations, such as those with disabilities, living in rural areas, or seeking to go into a particular profession (Kaufman, 2017; Kaufman et al., 2021). In 2016, a national survey showed that mentoring programs in the U.S. had implemented e-mentoring with only about 3% of participating youth (Garringer et al., 2017). However, with school closures and social distancing due to COVID, struggling mentoring programs began to see existing e-mentoring programs, such as iCouldBe, as an option.

iCouldBe uses a virtual multi-feature platform where low-income high school students complete “quests” to develop professional skills with support from an adult mentor. Mentees access the platform while at school in teacher-led mentoring sessions in which they connect remotely with their mentors; all interaction between mentors and mentees is virtual. The goal of participation in iCouldBe is for youth mentees to generate social capital, personal identity development and a sense of belonging, and ultimately to develop the building blocks of upward social mobility and income equality. iCouldBe focuses on developing skills and tools necessary for academic and future career success, including self-advocacy skills and a growing network of support (iCouldBe, 2022). In response to the COVID-19 pandemic, iCouldBe, with support from MENTOR (MENTOR, 2020), offered their platform as a free resource to mentoring programs that needed to transition to e-mentoring immediately as the pandemic lingered on (iCouldBe, 2020). After the spring and summer of 2020, iCouldBe continued to offer access to their platform to mentoring programs with some financial support for specific programs from external grants.

In general, the transition to e-mentoring during the pandemic posed many challenges for traditional in-person mentoring programs. Some programs and their participants lacked adequate technology and internet access to effectively implement e-mentoring (Kaufman, 2017). Others had challenges related to staff or parent buy-in. Still others were hampered by their own policies, which may have precluded contact between mentors and youth outside of program facilities (MENTOR, 2020). Even when technology, capacity, and adult motivation for e-mentoring aligned, many programs found that youth struggled with mental distress, such as depression and anxiety, due to social isolation (Kaufman et al., 2022). In addition to established e-mentoring programs, like iCouldBe, mentors and mentees used other methods of virtual communication to stay in touch, such as text messaging and FaceTime (Kaufman et al., 2022).

While other research looked at the immediate impact of the pandemic on mentors to youth (Kaufman et al., 2022), little is known about how mentoring programs handled the transition from in-person to e-mentoring during a time of crisis. It was incumbent on programs to maintain continuity in the mentoring relationships already established despite the isolation necessitated by the pandemic. This study aimed to inform e-mentoring practice in a post-COVID world through identification of what did and did not work well during the transition to e-mentoring during a time of crisis. We explored the experiences of mentoring programs during the first year of the pandemic and specifically their shift to e-mentoring during COVID.

## 2 Methods

### 2.1 Setting and participants

This descriptive qualitative study used online focus group discussions (FGDs) and an online pre-discussion program characteristics form (e.g., program type (one-to-one, group, peer; school-based, community-based), any special youth populations served, approximately how many mentees does the program serve). Eligible participants included mentoring program directors, match coordinators, and other pertinent program staff from youth mentoring organizations in the U.S. that used the iCouldBe platform during the Spring/Summer of 2020 or the Fall/Winter of 2020–2021. Participants met online for a 90-min FGD using Zoom video conference software.

### 2.2 Recruitment

MENTOR and iCouldBe led recruitment by emailing relevant organizations about the opportunity to participate in this study. Interested participants completed an eligibility screener and then, if eligible, an online consent form, both through Qualtrics, before participating in an FGD. Participants signed up for available times and then attended the appropriate FGD. Up to two people from each participating mentoring program were eligible to join the study.

### 2.3 Data collection

The research team prepared an FGD guide to provide prompts for the moderators to follow during the FGDs. This guide included questions about the organizations' experience with e-mentoring, such as how the pandemic affected the participants' mentoring programs (staffing, funding, mentee/mentor engagement, relationships with schools/community resources); the program's experience with e-mentoring before the COVID-19 pandemic (virtual communication between mentors and mentees, e-mentoring platforms); the program's technical support staff, if any, before and during the pandemic; if the program had support from within the organization for e-mentoring; and if the program had support from families, mentees, and mentors for e-mentoring. The guide also included questions about the programs' experiences using e-mentoring in general as well as using the iCouldBe platform specifically. Participants were asked to discuss any advice they would share with other programs about transitioning to e-mentoring as well as any requests they would make to the broader mentoring field regarding ways to make the transition to e-mentoring a smoother process. (See Supplement for full FGD guide.)

All FGDs occurred during April 2021. Two moderators (DL, KW, TA) led each FGD, asking the prepared questions and prompting for deeper explanations, when necessary. For two scheduled FGD sessions, only one participant was in attendance for each. These sessions were conducted as individual interviews but followed the same procedure and question guide as the FGDs.

### 2.4 Data analysis

FGDs were audio-recorded and transcribed; transcriptions were uploaded to Atlas.ti (version 9.1.3). Prior to coding, a codebook was created based on the FGD guide and the study objectives (KW). Two research team members (KW, AB) coded the data independently, using a deductive approach with pre-determined codes while also remaining open to adding or removing codes as the coders felt necessary. After each FGD was coded, the coders met to compare and discuss any discrepancies. When necessary, discrepancies were discussed until agreement was reached and were then adjusted by each coder individually. After all transcripts were coded, a reliability check was performed with 93.3% agreement (Creswell & Creswell Baez, 2021; Ravitch & Carl, 2021).

Thematic content analysis was performed by three research team members (KW, MS, TA) using a phenomenological approach. Transcripts were read, and each team member reviewed the coded data independently; common themes were developed through team discussions. Organizing the data into themes helped establish relevance and meaning. Reviewing the data multiple times and engaging in consistent team discussion provided an opportunity for confirming significance and understanding relationships in the data. Through this process, we also discovered discrepancies within our understanding of the data that we then could review and discuss to explain any disconnect. We analyzed the data set as a whole and then separated the data by Spring/Summer and Fall/Winter; by urban, suburban, and rural locations of the youth populations served; and by school-based and community-based programs to find any distinct themes within these groups. The research team continued to meet to discuss interpretation of the data and its relevance to the broader mentoring field (Creswell & Creswell Baez, 2021; Ravitch & Carl, 2021).

This study was approved by the Institutional Review Board at [author's university].

## 3 Results

### 3.1 Organizational profiles

**Fig. 1:**
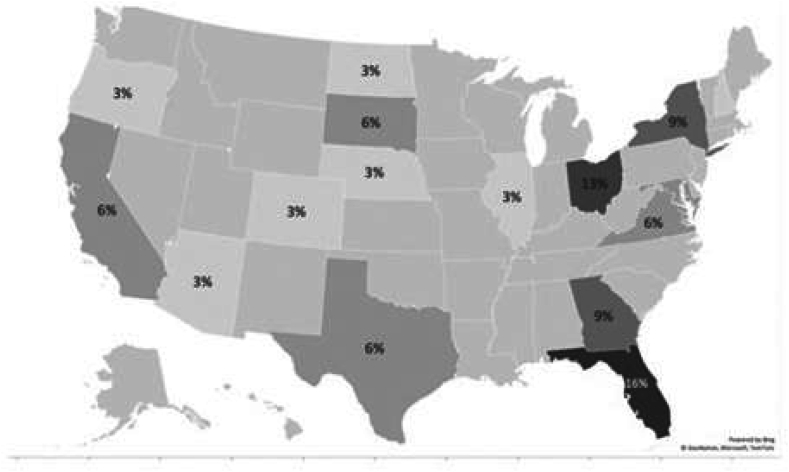
FGD representation of mentoring programs across the United States.

We conducted seven FGDs with 23 participants in total, representing 20 mentoring organizations that covered rural, suburban, and urban areas of the U.S. (Fig. 1) Most programs had one participant join the study; three programs had two participants join. More than half (60.87%) of participants came from small organizations with a staff of less than five people, followed by organizations with a staff of less than ten (17.39%). For the most part, organizations had a long history of about 20 years serving youth. Almost all participants were coordinators or managers/directors of their mentoring programs so only a low percentage of these participants worked directly with mentors and mentees (Table 1).

**Table 1:** Participant program characteristics.

Programmatic specifics	n = 23 (%)
*Number of staff*	
<5	14 (60.87)
5–10	4 (17.39)
11–50	3 (13.04)
51 or more	2 (8.70)
*Length of time in operation*	
1–5 years	2 (8.70)
6–10 years	2 (8.70)
11–20 years	9 (39.13)
More than 20 years	10 (43.48)
*Type of mentoring*^a^	
One-on-one	22 (95.7)
Group	9 (39.1)
Peer	1 (4.3)
School-based	14 (60.9)
Community-based	14 (60.9)
Corporate	4 (17.4)
*Grade level (based on age)*^a^	
Elementary school	15 (65.2)
Middle school	21 (91.3)
High school	22 (95.7)
18 years and over	3 (13.0)
*Programming for specific populations*^a^	
Male	7 (30.4)
Female	9 (39.1)
Transgender/non-binary	0
Sexual minority youth	2 (8.7)
Justice-involved youth	10 (43.5)
Rural youth	8 (34.8)
Urban youth	6 (26.1)
Youth of color	17 (73.9)
Indigenous youth	6 (26.1)
Gifted and talented youth	4 (17.4)
Immigrant/refugee youth	9 (39.1)
Youth with disabilities	7 (30.4)
Youth affected by the opioid crisis	4 (17.4)
Opportunity youth	7 (30.4)
Youth in foster care	13 (56.5)
Other (bereaved military children, youth experiencing homelessness, youth with increased risk factors, youth facing systematic barriers to employment)	1 each (4.3)

a Programs can select more than one option.

Most programs with FGD participants (95.7%) used one-on-one mentoring, but more than one-third of them (39.1%) also implemented group mentoring as a complement. An equal number of programs were school-based (60.9%) or community-based (60.9%), with a small percentage working through corporations (17.4%).

Our qualitative analysis uncovered the following themes, described in detail below:

1) experiences with e-mentoring, specifically in relation to virtual communication methods used by matches and activities created to encourage interaction between mentors and mentees;
2) challenges of the COVID-19 pandemic, including a loss of participation due to difficulties faced by mentors and mentees and capacity concerns within programs;
3) unexpected positives of the COVID-19 pandemic, such as expanding the program's reach through e-mentoring;
4) technological support, and the overall need to increase support and capacity; and
5) advice for programs and the mentoring field regarding the implementation of e-mentoring.

### 3.2 Experiences with e-mentoring

“For them [mentees] not to feel isolated, that ‘I’m just in this little silo by myself with my computer’ … has been the most effective part.” (Virginia)

**Fig. 2:**
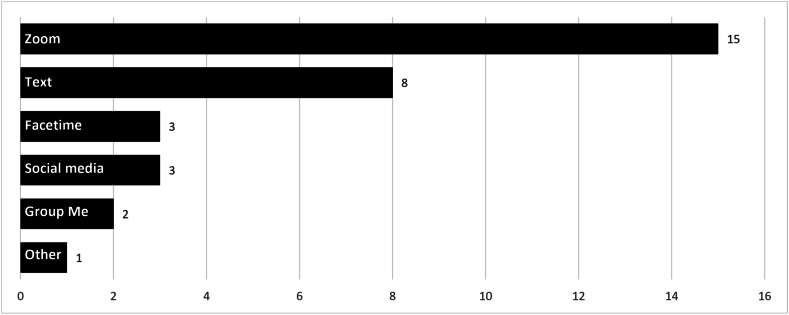
Virtual methods of communication used by mentors and mentees “Other” includes: Facebook Messenger, WhatsApp, phone, email, Skype, Microsoft Teams, and Google Meet.

Participants explained that mentors and mentees in their programs connected using many different forms of virtual communication. The most common ways to connect were through Zoom and text, followed by Facetime and social media (Fig. 2). Mentors and mentees who already used virtual means for connecting before the pandemic began continued to do so with ease. Participants indicated that iCouldBe was not often used as a means of connecting between mentors and mentees during this time.

It was easier for them to just text each other or to use whatever communication tool they were using previously. (South Dakota)

Programs came up with a variety of creative and fun opportunities for virtual connection. For example, participants described online scavenger hunts and art activities that mentors and mentees could do together. Some people also talked about playing online video games together. Geocaching was a method used to get mentees outside while still maintaining a safe distance. Some programs created interactive group activities. For example, one participant described a program-wide snowperson contest, during which everyone built a snowperson and shared a picture, and then voted on which was the favorite. Another participant explained their teen cuisine program—a program that met over Zoom and included cooking, nutrition, exercise, and the ability to add creativity to shared recipes. Innovative ideas such as these kept mentees and mentors engaged with each other and gave them all something to look forward to during the quarantine.

Our [teen cuisine] finale was yesterday, and they were able to make individual smoothies … it has probably been one of the most successful ways that we’ve had because it has been so interactive and they’re able to see the results of the work that they’ve done and they’re able to share it … We’re going to continue that program. (Virginia)

### 3.3 Challenges of the COVID-19 pandemic

“But you know, we manage, we are in survival mode.” (Georgia)

***Mentors and mentees:*** The COVID-19 pandemic brought a new set of challenges to the youth mentoring field. Participants described a loss of participation with at least 20% of both mentors and mentees. Some mentors showed little interest in e-mentoring or virtual connection with mentees at all. Others had to juggle personal changes, such as working from home, having kids at home doing virtual school, or job changes, and could not find the time or energy to continue their mentoring relationships.

Some mentors have been unable to meet because their job has changed or all of a sudden they’ve got kids at home … so those pairs are just kind of on hold. (Illinois)

We lost a couple mentors because they feel like the virtual mentoring is not for them … We’ve lost other mentors who don’t feel comfortable with being outside with the kids, so we have both sides right there. (Maryland)

Participants described mentees as having an overall lack of motivation to connect virtually with mentors. Many mentees were not interested in engaging over Zoom due to screen fatigue, especially among urban youth. Some mentees had to work during the day while also attending virtual school on their phones; connecting with a mentor beyond these responsibilities was not a high priority. In addition, some mentees did not have consistent access to devices or the internet, creating an additional burden for virtual mentoring relationships. Some mentees simply did not respond to their mentors’ attempts at connecting, and many pre-pandemic matches with relationships that were not as strong ended during the pandemic. Further, COVID restrictions offered little opportunity for community recruitment of new match participants, and school-based programs were overburdened with virtual schooling and prioritized this over mentee recruitment.

We’ve seen a lot of students that are working during the school day and going to school on their phone while they’re working … We’ve both seen and heard from our students that there’s a greater need to step in and provide for the family … I think that’s at least part of the explanation for why our numbers have dropped the way that they have, especially on the high school side. (Georgia)

Not everybody has a computer, and access to reliable Internet was a big challenge. We have families with multiple kids, and the Internet that they have wasn’t enough to cover the needs of those computers. (Ohio)

***Mentee families:*** Participants discussed difficulties that arose due to individual mentee family struggles, as many families were in survival mode. Some faced financial or technological difficulties; some shifted to working from home with children also doing school virtually; some families were living in isolation, lacking support. Programs found it hard to connect with parents, many of whom felt overwhelmed. When parents did communicate with programs, many felt uncomfortable with e-mentoring, mostly due to a lack of knowledge about this kind of programming. Parents of new mentees that programs took on since the start of the pandemic were more supportive of e-mentoring opportunities.

It was really difficult when we pivoted to all virtual because you need a lot of parent support for that to happen … A lot of the kids in our program come to us because they’re facing a lot more risk factors, they’re not getting a lot of support at home already so trying to add that was difficult. (Nevada)

At least 75% of the families or the parents were a little bit worried about [e-mentoring] because they don’t understand the platform … There was a little bit of resistance. (Maryland)

***Programs:*** Programs themselves also faced many difficulties due to the pandemic. Participants discussed a loss of staff, a lack of training on virtual programming for the staff they retained, and organizational financial concerns. Programs also described an overall lack of human resource capacity. For example, some programs had staff members taking on multiple roles or one person running the entire program. Some staff members had to shift from full-time to part-time hours due to financial concerns. Some programs faced issues with having enough mobile devices and struggled to provide devices to all their mentees and mentors who needed them. Urban programs, in particular, expressed a concern surrounding difficulties finding mental health support for mentees who needed it.

Without the staff capacity and without the device capacity, the challenges make it difficult when programs already feel overwhelmed with a pandemic. (New Hampshire)

A significant amount of our funding comes from fundraisers and galas and in-person events that we didn’t have the capacity to do during the pandemic … so our funding has definitely shifted, and our budget has almost halved. (California)

The pandemic introduced a new set of safety concerns and requirements for traditional mentoring programs. E-mentoring and the use of the internet often required an added layer of background checks for mentors in addition to requirements for in-person mentors, although due to capacity issues, some programs explained they ended up removing a layer of background checks instead. In addition, coordinators had to take on extra work as they felt they needed to monitor all Zoom calls. As pandemic guidelines relaxed over time, programs also had to create a new set of policies and procedures to safely allow for some in-person interactions.

We have to add in another layer to get permission from the families to make that [virtual] connection. (Georgia)

Our funders and our board had a waiver developed … and if all parties were comfortable, they were able to sign the waiver and see each other in person, following proper safety protocols … Our match support specialists are calling once a month, talking to the parent, the child, and the mentor just to make sure everyone is comfortable, no one’s in a bad situation, you’re OK with meeting, making sure they’re following safety measures. (Florida)

Participating programs struggled to follow the health guidelines the pandemic required, particularly rural programs from the Spring/Summer cohort. Some participants complained about a lack of leadership from their states, requiring them to take the time to figure out what guidelines they should follow. All participating programs stopped meeting in person, at least for a period of time, and they had to scramble to figure out what they could do to replace their in-person programming. Some programs talked about how COVID safety guidelines presented barriers to making new matches, especially when mentors wanted to meet online for safety reasons and mentees had no interest in additional virtual activities, as discussed above. School programs expressed frustration at the fact that volunteers were not allowed in schools, even after students returned to in-person learning. Some participants who ran peer mentoring programs described how difficult it was to get mentors and mentees in school on the same days when schools were operating under a hybrid format. The changing guidelines created confusion and obstacles for many mentoring programs.

Because we didn’t have that strong leadership from the State, we were really kind of just left to our own devices, and a lot of the decisions about what would be safe for the program fell … right on my shoulders. (South Dakota)

Our elementary kids came back for three weeks in October, November, about a third of them came back, then everything shut down again. In January, about a third of our students came back again … It’s extremely hard because it changes all the time. (Illinois)

### 3.4 Unexpected positives of the COVID-19 pandemic

“We just kind of kept stressing that no matter how you're doing it, no matter what the mode is, the important things about the relationship still matter. You still need to be expressing care and challenging growth and providing support.” (South Dakota)

The COVID-19 pandemic created a multitude of difficulties for mentoring programs. That being said, participants also noted a number of unexpected positives that came out of the pandemic. Participants noted benefits related to mentors and mentees as well as to the programs themselves.

***Mentors and mentees:*** The pandemic provided an opportunity for mentoring programs to find confidence in the ability of mentors and mentees to adjust to new and trying circumstances. Strong, pre-pandemic relationships remained solid and successfully shifted to using all virtual forms of communication, despite the struggles they may have faced.

Pairs that were really solidified and well-matched before this happened managed to find ways to still connect. (Nevada)

The mentors were really excited because when school ends in June, that mentor doesn’t talk to that student again until October, so all this time is lost. They were so excited to have an ability to catch up with them [over the summer]. (California)

The advent of e-mentoring expanded opportunities for mentees to connect with mentors from outside of the local area as well as mentors who did not have the ability to meet in person. By expanding their reach in this way, programs could more intentionally match mentees with mentors who better fit their specific needs.

The pandemic has allowed us to look at geography a lot differently, and one of the silver linings for me is I can really even drill down further on common interests and personality traits and, like, the sameness associated with some of the match connections I’m making. (New York)

We were able to start to reach … a whole population of mentors who had not been able to engage yet with us because of their job. They were not able to leave for an hour every week to go to the school and meet in person. Where now we’re able to see that there are parts of the adult mentoring professional world population of people who want to be able to mentor but can’t leave work or they live too far away from the district that they wanted to be connected with, and so e-mentoring was a really positive thing for them. (Ohio)

One participant who represented a program that worked with people with disabilities described the unexpected beauty of e-mentoring for this population as it created an opportunity for the mentee to begin a mentoring relationship without any preconceived notions in relation to their disability. Mentees were approached by mentors with the same expectations as mentees without physical or learning differences.

[This was] the first time where an adult had no expectations for them that were not based on some characteristic that they had no say, no choice in; that they were being paired with mentors who are adults who were interested in seeing them get through … [There was] a list of expectations about what was to be done, but nobody said, ‘Oh, you have a disability, so as long as you do something, I don’t care.’ (New York)

***Programs:*** Participants explained they were very pleased with their programmatic shifts to virtual processes. They used the pandemic as an opportunity to focus on meeting specific programmatic goals that had previously remained on their to-do list. For example, some programs updated their websites; some updated their trainings and transitioned them to a virtual format; others began using software to support mentoring operations. Many programs also shifted their onboarding to an all-virtual process, with interviews, screening, and matching all conducted remotely. Participants found this method to be easier and faster than their previous in-person onboarding process. Overall, staff at these programs experienced a much-needed improvement in technology competency.

We had always had e-mentoring in our plans, but the pandemic forced us to kind of ramp that up and allowed me to kind of put the other things aside to really focus on this. (New York)

There have been some really good things that have come out of it. We knew we needed to redo our website, we knew we needed to update some of our training and move more of it online … we’re doing more of those virtual … interviews and the screening part of the process, which has made it easier for our staff and our volunteers. (South Dakota)

The pandemic required creativity and innovation, and programs responded with the creation of new virtual programs. Participants felt excited about these successes and new opportunities, and most of them expressed a desire to continue with a hybrid mentoring model after pandemic restrictions had relaxed, allowing for in-person mentoring to return.

This year has been all about trying things and hoping that it works, and if it doesn’t, you know, pivoting again to see what else we can do. (Georgia)

We’re making it fun and interactive, so they’re sometimes used to playing solo games, but to do it in a group setting outside of class seems to be something that they were very receptive to. (Virginia)

Interestingly, we found that some programs had opposite experiences during the pandemic. Most notably, although many participants described their struggle with losing mentors and mentees, others talked about experiencing an increase in enrollment. These programs found that more mentees were able to participate in programming because e-mentoring removed the barrier of parents dropping kids off and picking them up. They also described new mentors as being more committed and consistent with their mentees.

I’m really impressed by the volunteers that have been coming to us throughout the pandemic … we’re noticing that the volunteers that are coming to us are more consistent and more committed than the volunteers that were coming to us before … and they’re really excited about mentoring, even if it’s on a screen. (Arizona)

We were able to recruit kids across our county. [Our] county is huge, and there’s some really, some very nice exclusive places … but there’s also other areas that are underserved and underrepresented kids, and we were able to recruit kids in those areas that typically wouldn’t be able to take a bus or get transportation to come to our office that now are able to participate. (Florida)

That being said, only four participants commented on increased participation, and only two clearly stated an increase in mentee and mentor participation. Both participants represented community-based programs that had an urban component, which may have contributed to the increase. Out of the other two who discussed an increase in participation, one participant described an increase in mentors and a decrease in mentees, while another described the opposite.

### 3.5 Technological support

“That’s [tech support] a dream come true, and one of the top things on our wish list.” (Virginia)

In terms of technology support, only one-sixth of respondents had a specific person in charge of technology. School-based programs often had consistent technology support and/or IT departments. Community-based programs were more likely to rely on tech consultants to provide support when needed, typically only for issues with devices or with the organization's intranet. Other programs described using board members, neighbors, past mentees, and other staff members who took on the tech support role without any official training. Participants clearly expressed a strong desire for more tech support.

I’m in the school district so we do have some [tech] support. (Ohio)

We do not [have technical support staff]; I stepped into those roles with really no formal training. (North Dakota)

One participant expressed happiness at the increase in the technology competence of families and mentors during the pandemic.

[One] pro I would say is that mentors and families became a lot more … their Internet competency certainly increased. (New York)

Only 8.7% of programs participating in the FGDs had e-mentoring in place prior to the pandemic, and most participants explained they had no support for the switch to e-mentoring. Instead, they learned how to use Zoom and already existing e-mentoring platforms while simultaneously creating a new mentoring program and supporting mentees and mentors during an unexpected crisis.

The biggest hurdle was, for me, getting the Zoom meeting set up, which the girls [the mentees] taught me. (Virginia)

I would say Zoom is a little, it’s supposed to be really straightforward, but it can definitely be tough when meetings expire or certain people know how to set it up to be a repeating meeting, kind of all those little things you see as you go along. (New Hampshire)

### 3.6 Advice for programs and for the mentoring field

“It feels like we’re all doing this on our own, but we’re all doing the same thing in our own corners, and it feels like there’s got to be a better way, especially in today’s world.” (Oregon)

***For programs:*** Participants offered advice for mentoring programs that were interested in incorporating e-mentoring. First and foremost, they suggested programs be patient and start small. The shift to e-mentoring takes time, but it is worth the effort.

Recognize that there are going to be things, that you think you’ve solved it, you know, you think you’ve got the perfect answer, and it’s probably not the perfect answer. Or you’re probably going to have to go through, you know, a few rounds to figure out what works for you and your participants and your families. (Georgia)

Maybe you start out with a smaller group and take it out to scale. (California)

Some participants recommended using e-mentoring with new matches instead of trying to shift previous in-person matches to a virtual format, especially regarding mentor/mentee relationships that had not yet developed a strong connection.

It seemed like a better fit for mentors that were brand new or new matches. (South Dakota)

Starting fresh is much more realistic than us, like, transitioning from in-person to e-mentoring. (Arizona)

To emphasize the importance of capacity, participants discussed the importance of hiring one staff person who is devoted to the e-mentoring aspect of the program. In addition, they suggested taking advantage of already existing opportunities (e.g., incorporating previously developed e-mentoring programs) and leaning in on technical assistance opportunities available to mentoring programs through MENTOR.

That’s really my heart’s desire, as we move into really exploring e-mentoring more, find someone who can be an e-mentoring program director because it’s just such a different world as far as the needs and the way you want to match people. (Ohio)

Lean in on the TA (technical assistance). You can’t buy that kind of support. (New York)

Where applicable, participants stated it is important to cater to people with language barriers or disabilities.

[It’s important to consider] the accessibility of the programming in terms of for people maybe who don’t speak English comfortably, don’t read and write English properly, but then also digital accessibility from the sense of like for people who have hearing impairments or visual impairments, thinking about how your programming can be flexible to the point that it’s universally accessible without being burdensome for the mentors or mentees. (New York)

***For the mentoring field:*** Participants also discussed ways in which the mentoring field could be more supportive of e-mentoring. Multiple people wanted to have access to a central website for mentoring programs and mentors that would provide support and ideas surrounding e-mentoring. Some people also wanted to include a component on a website for programs to connect and share with each other. Many participants felt they learned a lot from participating in this research and were interested in collaborating on a more consistent basis.

It feels like we’re all reinventing the wheel over and over and over again … we’re all facing similar things, we’re all showing up to the same conferences … and we’re all in the same spaces and sort of expressing the same needs and struggles, but having a home for that would be helpful. (Oregon)

I would love a website that we could send mentors to that has all the ins and outs of how to be a mentor. (Arizona)

Specific requests were made for more support in terms of mentor recruitment; e-mentoring knowledge, training, and webinars; and tasks that could be outsourced (e.g., data entry).

## 4 Discussion

This study summarizes the experiences of programs throughout the country in shifting from in-person youth mentoring initiatives to e-mentoring, including both the challenges and successes of this rapid pivot given the constantly shifting environment under the COVID-19 pandemic. FGDs revealed that many programs struggled with this immediate shift, although they also experienced some unexpected positives. Overall, e-mentoring worked well, especially for those mentor/mentee matches who used virtual apps, such as Zoom and texting. Mentoring programs expressed a need for improved technology support and a central website to support programs and mentors with e-mentoring. They also encouraged other programs interested in e-mentoring to take it slow and to be intentional with program planning.

Programs described having difficulties with both mentor and mentee engagement. This was consistent with a survey of adult mentors in the early days of the pandemic where one in five mentors did not have any contact with their mentees (MENTOR, 2020). Additionally, in support of this study's finding regarding a lack of interest in e-mentoring among older mentors specifically, recent studies reported that older adults admitted a lack of interest or confidence or a fear of using technology as barriers to virtual connection in general during the pandemic (Haase et al., 2021; O'Connell et al., 2022).

Programs in this study reported that mentees had a multitude of difficulties that created barriers to connecting with their mentors, including device or internet access. This struggle has been documented for American youth more broadly, specifically for low-income and Black students as well as those living in rural areas (Gaylord-Harden et al., 2020; Lai & Widmar, 2021; Stelitano et al., 2020; Vogels, 2020). A group of mentors from another study conducted in the U.S. at the beginning of the pandemic reported a similar finding for their mentees (Kaufman et al., 2022).

A few programs noted an increase in participation among mentees and/or mentors. The two programs who experienced an increase in both mentees and mentors both had an urban component. Though these numbers are too small to draw any firm conclusions, this increase may have been related to their urban location. Though urban programs experienced a number of struggles, our results suggested rural areas had greater difficulties with accessing technology than urban areas. As noted above, other studies have reported a similar difficulty for people living in rural areas (Lai & Widmar, 2021; Stelitano et al., 2020). This difference in access between urban and rural programs may have contributed to the greater potential for participation among urban youth and their mentors.

Participants in this study also reported that mentees did not want to connect with mentors because of screen fatigue. A program based in Philadelphia that supports immigrant youth had a similar experience during their transition to virtual programming due to the pandemic (Birkenstock et al., 2022).

Despite the decline in youth employment across the country during the pandemic (Desilver, 2021; Gould & Kassa, 2020), some participants in the current study reported that mentees were working during the school day, and some were attempting to attend virtual school on their phones at the same time. These additional burdens made it increasingly difficult for programs and mentors to reach their mentees. This discrepancy between the national trend for youth employment and participant reports of mentees working may be explained by a higher prevalence of low-income families with youth as mentees (Garringer et al., 2017; Raposa et al., 2019, Raposa et al., 2019), whose youth may have felt more pressure to work.

Programs in this study reported having a mixed experience with the pandemic, describing the many difficulties but also unexpected positive consequences of the shutdown, such as opportunities to connect mentees with more fitting mentors in different locations. This opportunity provided by e-mentoring has been highlighted previously; one international mentoring program successfully connects mentees with mentors from around the globe, primarily using technology as the main and sometimes only means of communication (Garringer et al., 2019). Other programs with an e-mentoring component, not specifically focused on youth, are able to create mentor/mentee matches based on similar interests, goals, values, and beliefs (Chong et al., 2020).

Despite free access to iCouldBe's platform, video chat and text were the main methods of virtual communication used by mentees and mentors, with Zoom used most. In a previous study conducted very early on in the pandemic, mentors reported using text as the main method of communication with mentees, with Zoom used less frequently (Kaufman et al., 2022). This change in choice of communication methods may be one example of how the pandemic has altered the way people communicate. This new hybrid way of bringing mentors and mentees together opens doors for creative and exciting pathways of support and positive impact.

Participants in this study requested centralized resources for best practices for e-mentoring and connecting with other program stakeholders. A similar idea was suggested by mentors early on in the pandemic, who requested ideas and resources for connecting with mentees as well as an online support group (Kaufman et al., 2022). Bringing the mentoring community together in a more cohesive way can reduce stress for program staff and mentors and can improve programmatic effectiveness through positive communication and support. This type of support system can be impactful not only for e-mentoring but for all types of mentoring.

## 5 Limitations

This study has a few limitations. Due to the nature of qualitative data and the small sample size, results are not generalizable beyond the participants’ programs they represented. Program participants were also recruited using a convenience sample (those included on a mailing list provided by MENTOR) and may not be representative of the true nature of mentoring programs across the U.S. Because almost all participants were program coordinators or managers/directors, it is likely they spoke more to the operational scope and needs of their respective organizations and less to the experience of working in the field directly where the immediate factors impacting mentor/mentee relationships are found. Future studies may include the perspectives of programmatic staff, mentors, and mentees to provide a more comprehensive understanding of how to pivot to e-mentoring during times of crisis and natural disasters.

Participants in this study were separated into two distinct groups, based on the timing of their use of iCouldBe: Spring/Summer 2020 and Fall/Winter 2020/2021. Some of the programs may have only just started using the platform shortly before the FGDs occurred. The results presented here do not express the program experiences with iCouldBe but rather their experiences with e-mentoring in general, which most participants agreed began fairly quickly after March 2020. We believe participants had experienced e-mentoring for similar lengths of time.

## 6 Conclusion

Although the data on e-mentoring remains limited, this study contributes to the literature with a picture of e-mentoring success even in the midst of a global crisis. Traditional, in-person mentoring programs successfully connected their mentors and mentees using virtual methods of communication, and many plan to continue to use e-mentoring alongside their in-person format. This transition was not easy, however, and programs currently interested in incorporating e-mentoring into their programming can heed the advice of those who made the transition during the crisis by taking it slow and being intentional. The mentoring field overall can augment the capacity of mentoring programs by introducing new and creative ideas for e-mentoring, providing training for best practices in e-mentoring, and connecting programs and mentors from any location.

## Funding

This research was funded by the 10.13039/100000865Bill & Melinda Gates Foundation. The views expressed are those of the authors and should not be attributed to the foundation.

## Declaration of competing interest

none

## Data Availability

Data will be made available on request.
