# Supplementary material for: From crisis to capacity: Lessons learned from youth e-mentoring during the COVID-19 pandemic
Source: Comput Hum Behav Rep. 2024 May;14:None. doi: 10.1016/j.chbr.2024.100400 (PMC11127795; doi:10.1016/j.chbr.2024.100400)
Supplement: Multimedia component 1 [file mmc1.docx]

Supplementary material: FGD guide

Focus Groups with VMP Beginners/Users (Aim 1)

*Please complete these background questions prior to starting the discussion.*

1. **Program name**
2. **Your title in the program**
3. **How many staff work for the program?**
   - Less than 5
   - 5-10
   - 11-50
   - 51-100
   - Over 100
4. **How long has your program been serving youth?**
   - 1-5 years
   - 6-10 years
   - 11-20 years
   - More than 20 years
5. **Are there any staff in charge of technology at your program?**
   - Yes
   - No
   - Don’t Know
6. **How many staff work directly with mentees/mentors/families?**
7. **Program Type (check all that apply):**
   - One-to-one
   - Group
   - Peer
   - School-based
   - Community-based
   - Corporate (mentors from a large company mentor local youth)
   - Other
8. **What format was your program prior to the COVID-19 pandemic?**
   - In-person
   - E-mentoring/virtual mentoring
   - Hybrid
9. **PRIOR TO THE START OF THE PANDEMIC, approximately how many mentees were in your program?**
10. **PRIOR TO THE START OF THE PANDEMIC, approximately how many mentors were in your program?**
11. **Approximately how many mentees are in your program NOW?**
12. **Approximately how many mentors are in your program NOW?**
13. **What age youth do you serve?**
14. **Do you have specific programming for any of the following youth (check all that apply)?**
    - Male
    - Female
    - Transgender
    - Non-binary
    - Sexual minority youth (gay, lesbian, bisexual)
15. **Do you serve any special youth populations (check all that apply)?**
    - Justice-involved youth
    - Rural youth
    - Urban youth
    - Youth of color
    - Indigenous youth
    - Gifted and talented youth
    - Immigrant/refugee youth
    - Youth with disabilities
    - Youth affected by the opioid crisis
    - Youth who were sex-trafficked/commercially sexually exploited
    - Opportunity youth
    - Foster care youth
    - Other
16. **What are the goals of your mentoring program?**
17. **When did you reach out to iCouldBe and/or MENTOR to learn more about joining the Virtual Mentoring Platform (VMP)?**
    - spring 2020 (April/May)
    - summer 2020 (June/July/August)
    - fall 2020 (September/October/November)
18. **How far did you get into the on-boarding process to join the VMP?**
19. email follow up from iCouldBe to determine readiness
20. meeting to discuss program details and VMP project scope
21. sign Partnership Agreements
22. onboarding of mentors/mentees
23. mentors/mentees used/are using the VMP

***Now I want to start by asking you questions about your experience with e-mentoring.***

1. How has the pandemic affected your program, if at all?
2. Did you have any experience with e-mentoring and/or virtual activities with youth **prior** to the start of the pandemic? For instance, did mentor and mentee pairs chat or Facetime between in-person meetings, or connect via their social media accounts regularly?
3. Does your organization have technical support staff, such as an IT person, or operating officer?
4. What was attractive to you about the Virtual Mentoring Portal (VMP)?
5. Do you feel your colleagues at your organization were supportive of the switch to e-mentoring? Why or why not?
6. Do you feel the families/mentors/mentees were supportive of the switch to e-mentoring? Why or why not?

***[For groups that started the on-boarding process but did not enroll mentors/mentees on the platform]***

1. How far did you get in the onboarding process? What challenges did you face in onboarding?
2. Did you do e-mentoring using other forms of technology?

***[For groups that completed the on-boarding process and enrolled mentors/mentees]***

1. How long did you use the VMP? Are you still using it?

***[For groups just starting to use VMP in fall/winter 2020]***

1. What strengths do you think e-mentoring will provide to your program?
2. Are there any challenges you foresee that may make it hard for you to be successful with e-mentoring?
3. What opportunities do you think e-mentoring presents for your program?

***[ALL GROUPS]***

1. What is the biggest piece of advice you have for programs that are interested in trying e-mentoring?
2. What would you like to see the mentoring field (MENTOR, researchers, technical assistance providers) offer to programs who want to try e-mentoring?
3. What else do you think we should know about mentoring programs that want to pursue e-mentoring?
4. Do you have any questions for us?
